# Supplementary material for: Determination of Floral Origin Markers of Latvian Honey by Using IRMS, UHPLC-HRMS, and 1H-NMR
Source: Foods. 2021 Dec 24;11(1):42. doi: 10.3390/foods11010042 (PMC8750591; doi:10.3390/foods11010042)
Supplement: Supplementary file 1 [file foods-11-00042-s001.zip › Supplementary figures.pdf]

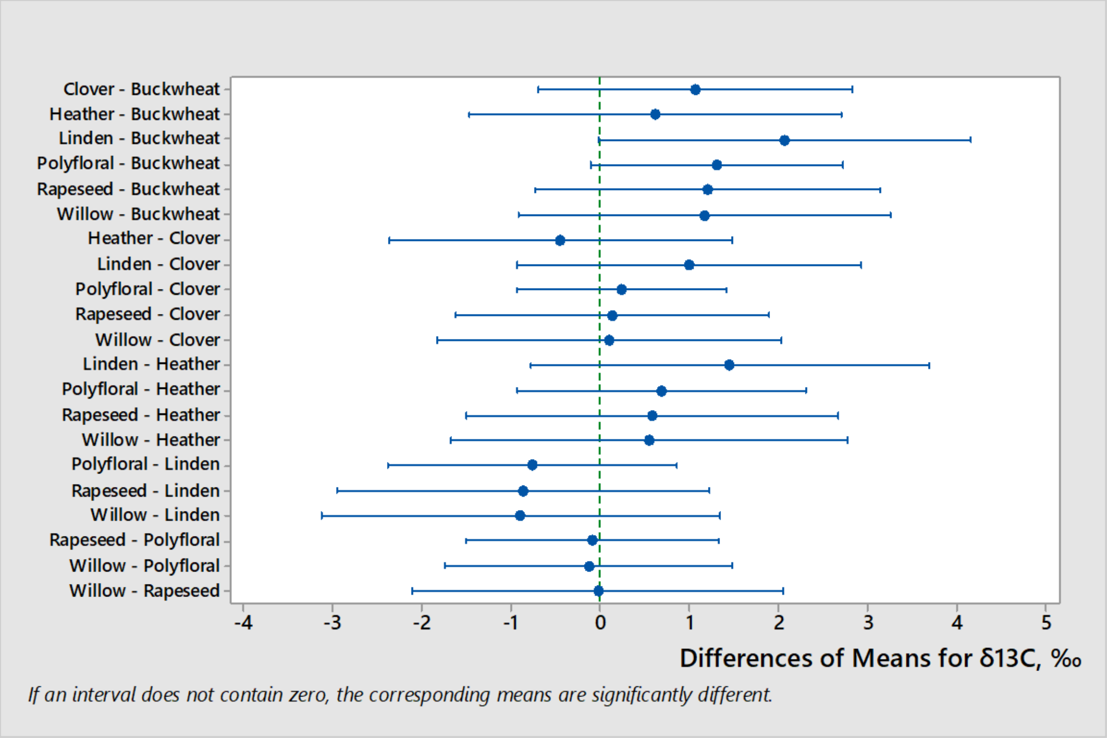

Figure S1. A graphical summary of ANOVA one-way test of statistically significant $\delta^{13}C$ value difference between monofloral buckwheat, clover, heather, linden, rapeseed, willow and polyfloral honey proteins using Tukey comparison of 95% confidence intervals.

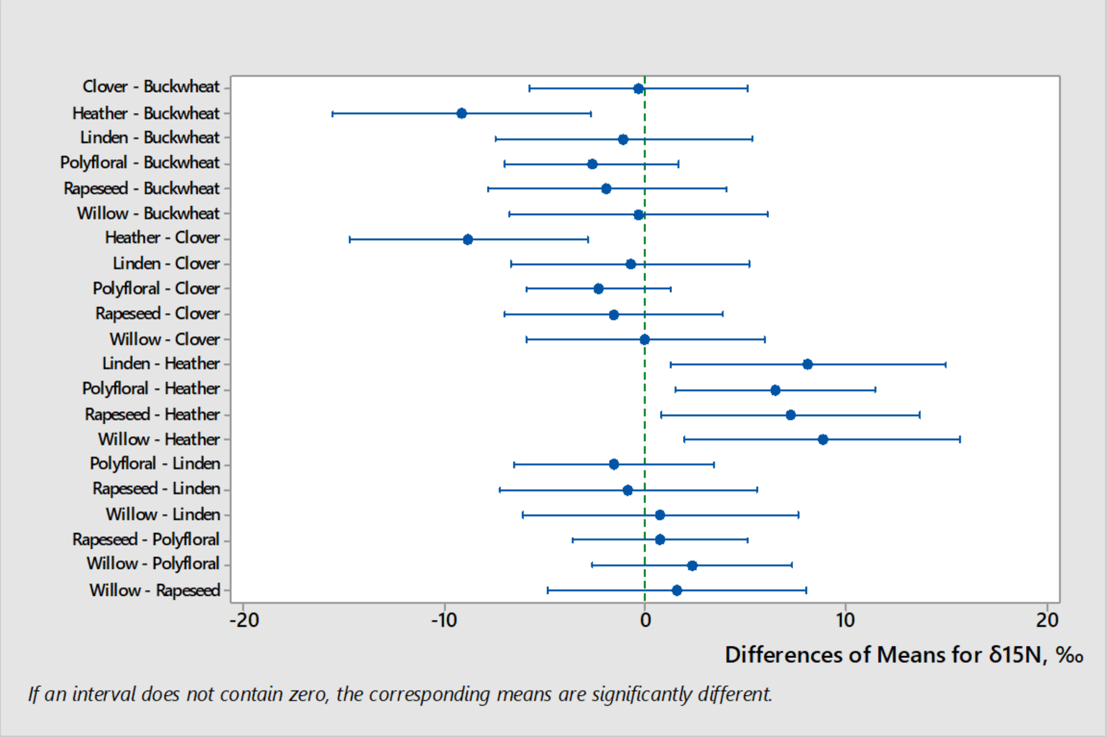

Figure S2. A graphical summary of ANOVA one-way test of statistically significant $\delta^{15}N$ value difference between monofloral buckwheat, clover, heather, linden, rapeseed, willow and polyfloral honey proteins using Tukey comparison of 95% confidence intervals.

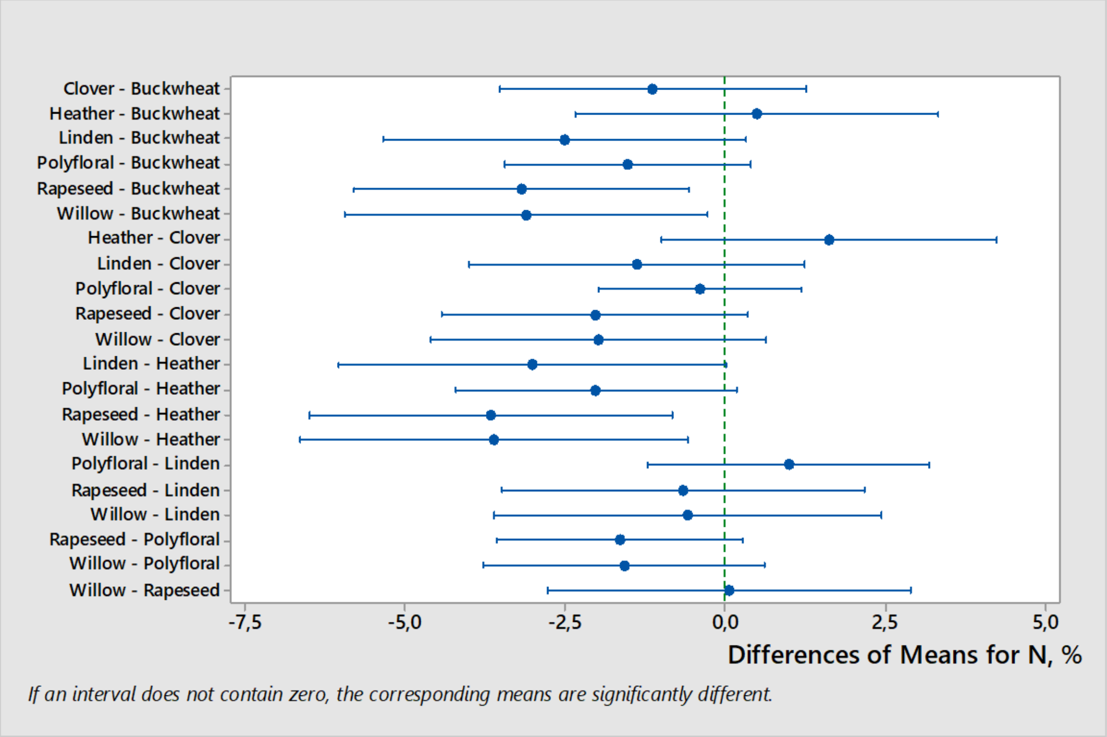

Figure S3. A graphical summary of ANOVA one-way test of statistically significant total N (%) value difference between monofloral buckwheat, clover, heather, linden, rapeseed, willow and polyfloral honey proteins using Fisher comparison of 95% confidence intervals.

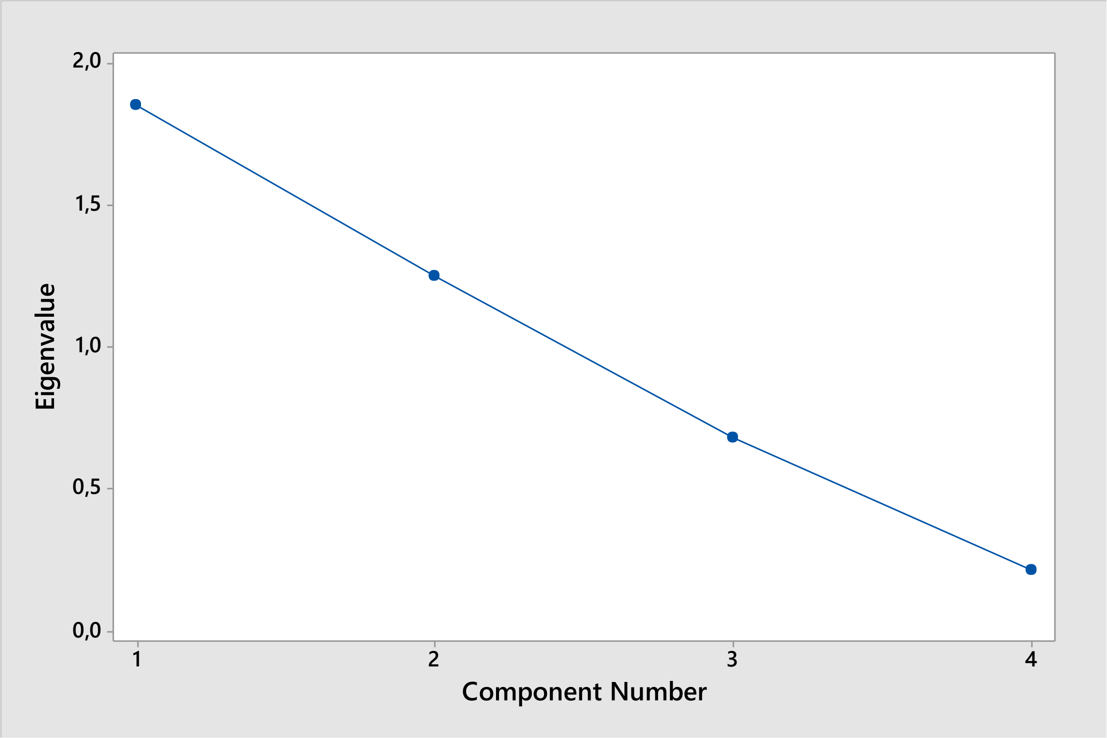

Figure S4. Scree plot of PCA eigenvalues of components. As input data variables were used $\delta^{13}C$, $\delta^{15}N$, total C & N in proteins.

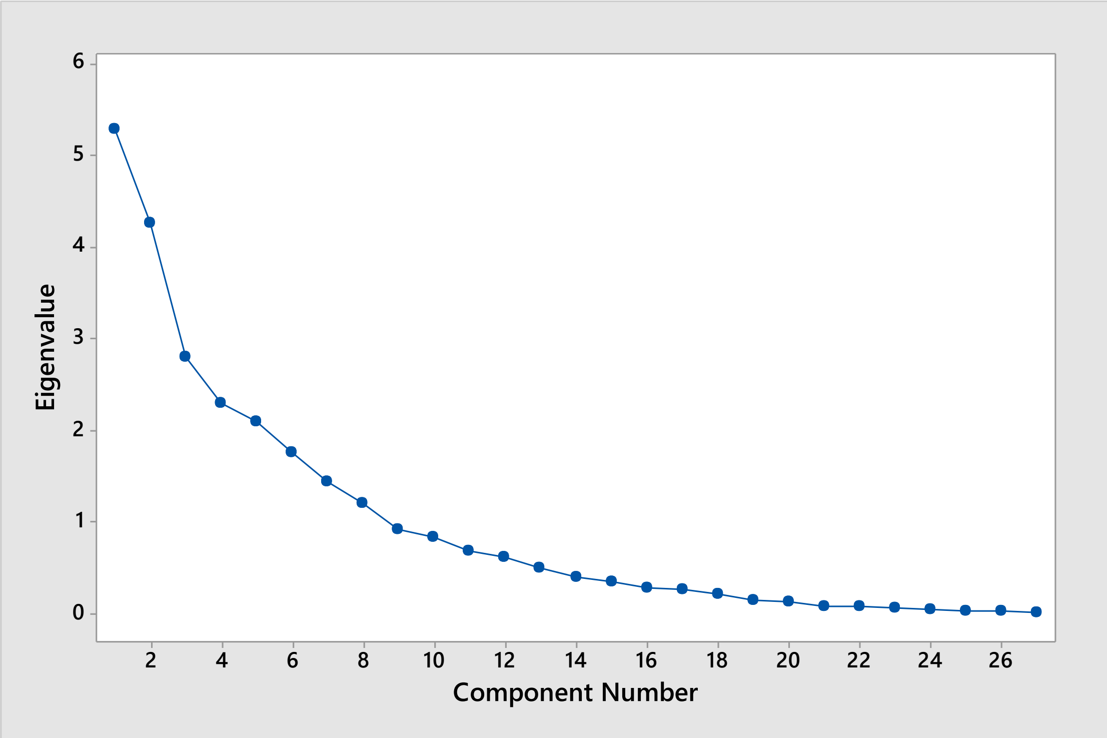

Figure S5. Scree plot of PCA eigenvalues of components. As input data variables are used from UHPLC-HRMS obtained concentrations of organic compounds.

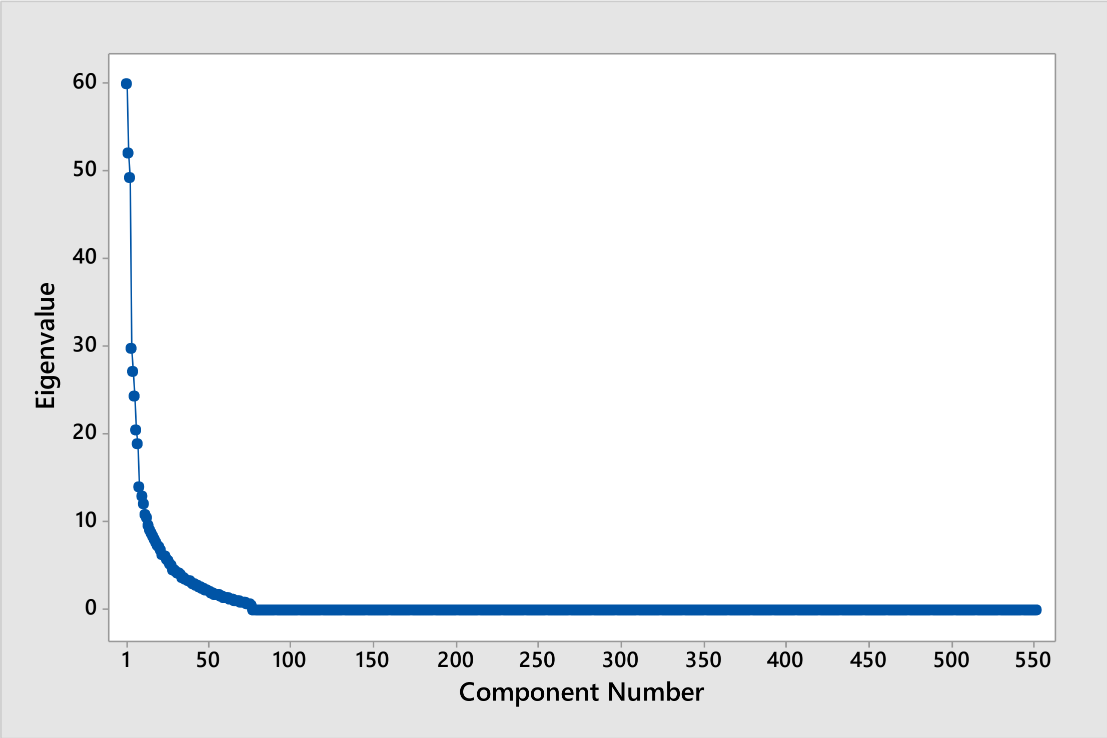

Figure S6. Scree plot of PCA eigenvalues of components. As input data variables are used binned ^{1}H-NMR spectra intervals 9.0-6.0 & 3.0-0.5 ppm with bin width 0.01 ppm.

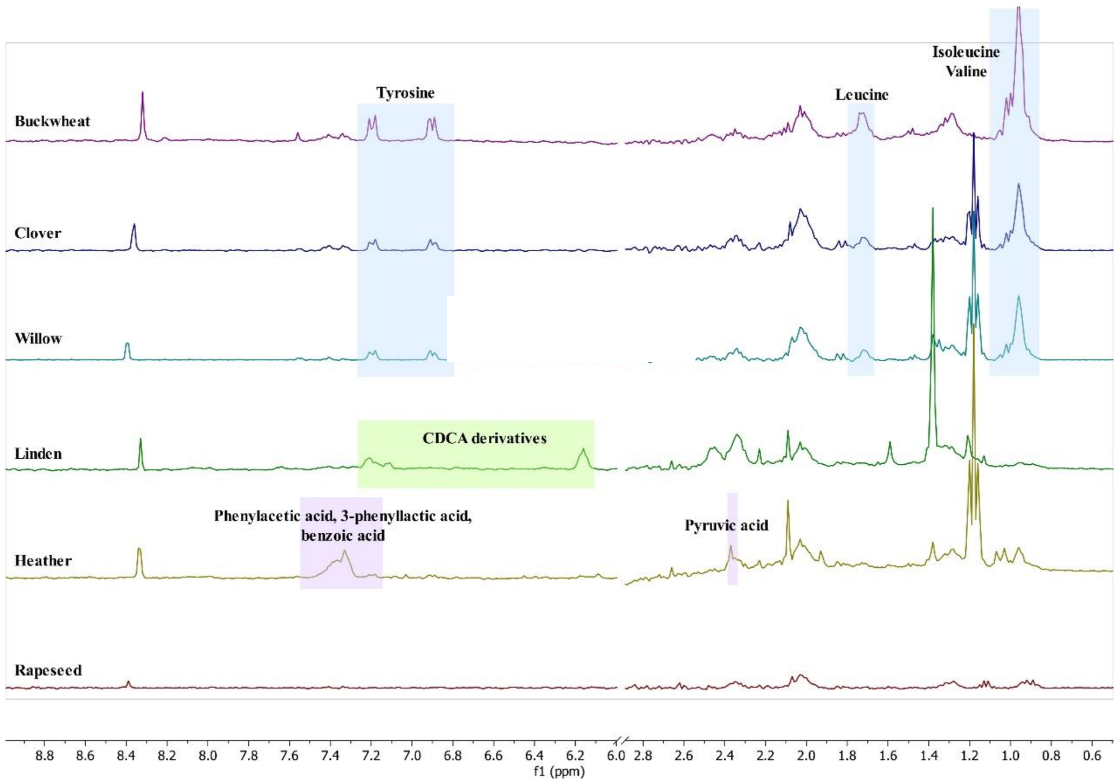

Figure S7. ^{1}H-NMR spectra interval (0.5 – 3.0 & 4.5 – 8.5 ppm) overlayed comparison of monofloral buckwheat, clover, willow, linden, heather, rapeseed honey.
